# A Quartet Network Analysis Identifying Mechanically Responsive Long Noncoding RNAs in Bone Remodeling

**DOI:** 10.3389/fbioe.2022.780211

**Published:** 2022-03-09

**Authors:** Jingyi Cai, Chaoyuan Li, Shun Li, Jianru Yi, Jun Wang, Ke Yao, Xinyan Gan, Yu Shen, Pu Yang, Dian Jing, Zhihe Zhao

**Affiliations:** ^1^ State Key Laboratory of Oral Diseases and National Clinical Research Center for Oral Diseases, West China Hospital of Stomatology, Sichuan University, Chengdu, China; ^2^ Department of Oral Implantology, Shanghai Engineering Research Center of Tooth Restoration and Regeneration, School and Hospital of Stomatology, Tongji University, Shanghai, China; ^3^ Institute of Engineering Medicine, Beijing Institute of Technology, Beijing, China; ^4^ School of Basic Medical Sciences, Chengdu University, Chengdu, China; ^5^ Department of Orthodontics, China Shanghai Ninth People’s Hospital, College of Stomatology, Shanghai Jiao Tong University School of Medicine, Shanghai, China; ^6^ National Center for Stomatology, National Clinical Research Center for Oral Diseases, Shanghai Key Laboratory of Stomatology, Shanghai Jiao Tong University, Shanghai, China

**Keywords:** mechanotransduction, long noncoding RNA (lncRNA), competing endogenous RNA (ceRNA), transcription factor (TF), bone, mesenchymal stem cells (MSCs), microRNA (miRNAs), force

## Abstract

Mechanical force, being so ubiquitous that it is often taken for granted and overlooked, is now gaining the spotlight for reams of evidence corroborating their crucial roles in the living body. The bone, particularly, experiences manifold extraneous force like strain and compression, as well as intrinsic cues like fluid shear stress and physical properties of the microenvironment. Though sparkled in diversified background, long noncoding RNAs (lncRNAs) concerning the mechanotransduction process that bone undergoes are not yet detailed in a systematic way. Our principal goal in this research is to highlight the potential lncRNA-focused mechanical signaling systems which may be adapted by bone-related cells for biophysical environment response. Based on credible lists of force-sensitive mRNAs and miRNAs, we constructed a force-responsive competing endogenous RNA network for lncRNA identification. To elucidate the underlying mechanism, we then illustrated the possible crosstalk between lncRNAs and mRNAs as well as transcriptional factors and mapped lncRNAs to known signaling pathways involved in bone remodeling and mechanotransduction. Last, we developed combinative analysis between predicted and established lncRNAs, constructing a pathway–lncRNA network which suggests interactive relationships and new roles of known factors such as H19. In conclusion, our work provided a systematic quartet network analysis, uncovered candidate force-related lncRNAs, and highlighted both the upstream and downstream processes that are possibly involved. A new mode of bioinformatic analysis integrating sequencing data, literature retrieval, and computational algorithm was also introduced. Hopefully, our work would provide a moment of clarity against the multiplicity and complexity of the lncRNA world confronting mechanical input.

## Introduction

Mechanic force is recognized as a crucial factor regulating cellular physiological properties and directing cell fate (Thompson et al., 2012; Hao et al., 2015). Particularly, as one of the most active organs of human body undergoing consecutive remodeling, bone is exposed to a dynamic mechanic environment and shows dependence on mechanical cues for homeostasis maintenance (Thompson et al., 2012; Petridou et al., 2017). Forces like strain, fluid shear stress (FSS), compression, and microgravity (MG) have been reported to regulate bone reconstruction (Thompson et al., 2012). Remarkably, signaling pathways responding to force as well as the final impacts on cellular fate after force application differ among types, duration, intensity, and other parameters of the mechanic stimuli, which has already been detailed by us before (Hao et al., 2015). Though the whole picture of how components of bone function during the process is far from clear, what can be sure is the involvement of bone-related cells such as osteoblasts, osteocytes, osteoclasts, chondrocytes, and mesenchymal stem cells (MSCs) of various origins, in forming an entwined concert both spatially and temporally. Notably, periodontal ligament cells (PDLCs) and periodontal ligament stem cells (PDLSCs), as the specific cells seeding in jaws, turn out to be highly sensitive to force and responsible for bone remodeling during orthodontic tooth movement, which make them ideal model for mechanical study (Huang et al., 2018).

Yet with an obscure landscape so far, the ways cells sense and react to those fickle and perplexing mechanical cues are extensively explored. To begin with, an organism would depend on the so-called “mechanosensors” to perceive and convert the information into cellular ones. Focal adhesions (FAs) and cytoskeleton, as well as membrane channels which contribute to force sensing, are mostly explored. FAs play as the crucial initiator in detecting and transducing extracellular matrix (ECM)-related biomechanical properties. The FA complex functions by having the integrin heterodimer at the cell surface to interact with the extracellular information and subsequently putting docking proteins to connect F-actin bundles (Qin et al., 2020). Hence, the cues induce the contraction of cytoskeleton, contributing to the opening of nuclear pores by direct contact with nucleus via the linker of nucleoskeleton and cytoskeleton (LINC) (Bouzid et al., 2019). Piezo1/2, as the calcium (Ca^2+^)-permeable channels, was proven to be another mechanosensor essential for bone homeostasis. *Via* the critical intracellular Ca^2+^ sensor Ppp3ca, Piezo1/2 would contribute to the dephosphorylation and formation of the NFAT-Yap1-Ctnnb1 transcription factor (TF) network. The TFs were then activated and showed nuclear translocation for downstream gene regulation (Wang L et al., 2020; Zhou et al., 2020).

Once mechanical cues are transmitted into cells, cascade signaling pathways and diversified molecules would be involved in the response. In recent years, noncoding RNAs (ncRNAs) have gained the limelight for their potent regulation effects. NcRNAs hold a share of almost 98% of all genomic outputs in humans, contributing to an extensive manipulation of the transcriptional activities. MicroRNAs (miRNAs), as the most extensively studied type of ncRNAs, have gained wide recognition for their mechanosensitive roles, which were detailed before (Chen et al., 2017; Yuan et al., 2017). Nonetheless, a force-related study on long noncoding RNA (lncRNA) was far from completed. As transcribed RNA molecules longer than 200 nucleotides in length, lncRNAs could transform into unique secondary conformation, enabling its direct binding with DNA, RNA, and protein and indicating its significant roles (Mercer and Mattick, 2013). Meanwhile, lncRNA has also been attested to be a ubiquitous regulator in diversified biological processes *via* multiple function modes (Ørom and Shiekhattar, 2013; Cech and Steitz, 2014). Significantly, the competing endogenous RNA (ceRNA) mechanism accounts for a great part of lncRNA-oriented research where lncRNAs would act as the “sponge” to hijack miRNAs from their binding with mRNAs, thus relieving the degradation or decoy effects of miRNAs on the targets (Kartha and Subramanian, 2014; Tay et al., 2014). Alongside lncRNA’s well-known function modes, the upstream regulation of its transcription controlled by TFs is another eye-catching project. Intriguingly, lncRNAs and TFs could form entwined crosstalk and even contribute to a feedback loop, considering that TFs, as one particular type of mRNAs, can be regulated by ceRNA interactions in the posttranscriptional level as well as regulate gene transcription after its translocation to the nuclear (Ji et al., 2020). Thus, networks illustrating ceRNAs and TF–lncRNA interaction could provide us with an overview of the lncRNA world from both upstream regulation and downstream effects (Alam et al., 2014; Ji et al., 2020).

**FIGURE 1:**
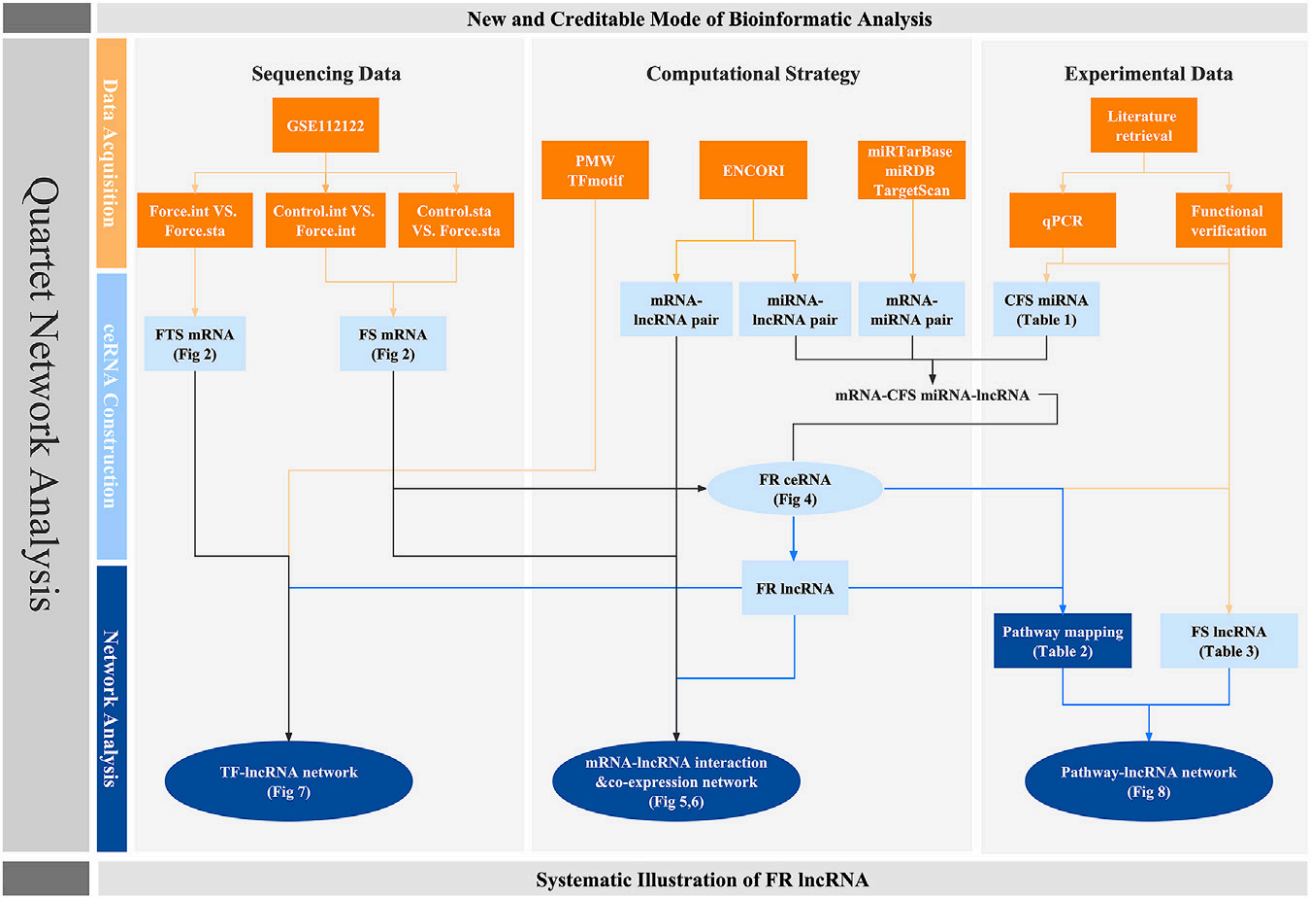
Workflow of this research. Orange squares stand for the database and evidences on which our research is based. Light and dark blue colors represent the crucial factors and networks predicted though bioinformatic strategy, respectively. Circles are used to highlight the quartet network of lncRNAs.

Moreover, there are well-documented compelling proofs that lncRNAs participate in the chondrogenic and osteogenic differentiation of MSCs and beyond, indicating the significance of lncRNAs in biophysical processes of bones (Huang et al., 2019; Ju et al., 2019; Sun et al., 2019; Wang H et al., 2020). Emerging evidence postulated that lncRNA could react to force condition and H19 was already proven to sponge miR-138 to regulate hMSC activity via FAK in a tension-induced way (Wu et al., 2018; Liu H. et al., 2019). A new TMSB4 pseudogene lncRNA related to mechanical stress (lncRNA-MSR) would hijack miR-152 to control TMSB4 expression in cyclic tensile strain (CTS)-induced regulation of chondrocytes and promote cartilage degradation (Liu et al., 2016). However, the systemic roles of lncRNAs in a mechanosensitive way remain obscure. Thus, aiming at highlighting an lncRNA-focused network, in which bone cells and their precursors would possibly activate to respond to the mechanic stimuli, we first explored a Gene Expression Omnibus (GEO) dataset for force-sensitive mRNA (FS mRNA) identification. Gene Ontology (GO) analysis was exploited for deeper knowledge of both the general and unique mechanisms that cells utilized for force responses. Instead of utilizing miRNA sequencing data which lack potent evidences, we screened out the creditable force-sensitive miRNAs (CFS miRNAs) based on reported research including qPCR and function experiment data to ensure reliability. Force-related lncRNA (FR lncRNAs) and ceRNA (FR ceRNA) networks were then predicted and constructed. Furthermore, to explicate the functional mode of FR lncRNAs, we displayed the direct interaction as well as the co-expression among FS mRNAs and FR lncRNAs. Crosstalk among TFs and lncRNAs by TF binding site (TFBS) prediction and identified several lncRNA-TF loops, demonstrating the potential upstream regulation on lncRNAs. Last, based on the analysis of our networks, we deciphered and summarized the potential mechanotransduction signaling pathways that FR lncRNAs may participate in, which were later integrated with the established data to build a pathway–lncRNA network. The network-centered strategy could highlight potential interaction and new roles of identified factors while suggesting a promising field worth exploring (Figure 1).

## Methods and Materials

### High-Throughput Sequencing Data Acquisition and Differentially Expressed Gene Analysis

Systematic searching of datasets concerning mechanical conditions was carried out in the GEO database (http://www.ncbi.nlm.nih.gov/geo/) for inclusion of FS mRNA. GSE112122 (https://www.ncbi.nlm.nih.gov/geo/query/acc.cgi?acc=GSE112122) was finally chosen based on its ideal cell type, experimental strategy, and data quality. Raw data of the gene expression profiles of GSE112122 were downloaded for analysis, and several grouping strategies were developed. Namely, intermittent force group analysis applied differentiated expression comparison based on data from control (GSM3058133-3058135) vs. intermittent force (GSM3058136-3058138). Similarly, static force group analysis utilized strategy as control (GSM3058139-3058141) vs. static force (GSM3058142-3058144). To explore the deviation of mRNA profiles caused by force types, intermittent force (GSM3058136-3058138) and static force (GSM3058142-3058144) are set as grouping modes. The Empirical Analysis of Digital Gene Expression Data in R (edgeR) package (Robinson et al., 2010; McCarthy et al., 2012) (http://www.bioconductor.org/packages/release/bioc/html/edgeR.html) was used to screen out differentially expressed mRNAs (DE mRNAs). Gene ID was converted to gene name and noted for gene type based on the Ensembl database (http://asia.ensembl.org/index.html). Only genes with type annotation as “protein coding” were selected for further analysis, which ruled out several noncoding RNA expression profiles with low confidence. The *p*-value was calculated with *t*-test and adjusted for multiple testing using the false discovery rate (FDR) method. A threshold was set as FDR < 0.05 and |log2foldChange (FC)| > 1 for DE mRNA identification. The Pretty Heatmaps (pheatmap) package was exploited to visualize the differences. Top 50 genes with the highest values of |log2foldChange (FC)| were chosen for heatmap creation. All analyses were performed with the R 3.5.0 framework. Intersection of DE mRNAs from intermittent force group and static force group analyses contributed to the list of FS mRNA. DE mRNAs gained from the comparison between profiles of intermittent force and static force were named as force-type-sensitive mRNAs (FTS mRNAs). VENNY 2.1 (https://bioinfogp.cnb.csic.es/tools/venny/index.html) was explored for visualization. Functional annotation for DE mRNAs was conducted based on GO analyses. All functional clustering was assessed by the clusterProfiler package (Yu et al., 2012) (http://www.bioconductor.org/packages/release/bioc/html/clusterProfiler.html). The Enrichplot package (http://www.bioconductor.org/packages/release/bioc/html/enrichplot.html) and ggplot2 package were implemented to visualize the enrichment results. Significant GO terms were identified with a cutoff of *p* < 0.05.

### Exploration and Pathway Analysis of CFS miRNAs

To identify reliable miRNAs which are sensitive to force among bone cells, we explored and collected the miRNAs which were verified to act in a mechanosensitive way via functional experiments or displayed certified different expression profiles in a force-related situation by qPCR. Cell types included in our work are confined to MSCs and their derivations in bone tissues. Research made before 2010 was excluded, as well as those without highly reliable evidence. CFS miRNAs identified in species other than *Homo sapiens* were further analyzed in miRBase (Kozomara et al., 2019) (http://www.mirbase.org/) for validation of their conservation among species, especially the similarity of sequences compared to their counterpart hsa-miRNAs. CFS miRNAs were uploaded into DIANA-mirPath v3.0 (Vlachos et al., 2015) (http://snf-515788.vm.okeanos.grnet.gr/) for KEGG analysis.

### Construction of FR CeRNA Network and Predication of FR LncRNAs

Aiming at constructing the potential FR ceRNA network, we first screened out the lncRNA targets of CFS miRNAs, utilizing ENCORI (the Encyclopedia of RNA Interactomes) (Li et al., 2014) (http://starbase.sysu.edu.cn/) for prediction. The analysis was based on human genome and hg19 assembly, and the cutoff value was set as CLIP Data ≥ 3 (high stringency). Then, CFS-miRNA-targeting mRNAs were also predicted based on the intersection of datasets from miRTarBase (Chou et al., 2018) (http://mirtarbase.mbc.nctu.edu.tw), miRDB (Chen and Wang, 2020) (http://www.mirdb.org/), and TargetScan (Lewis et al., 2005) (http://www.targetscan.org/mamm_31/). MiRNA–mRNA pairs that gained validation from all three datasets were included. The predicted pairs were then intersected with the FS mRNA. Only mRNAs which were both force sensitive and predicted as miRNA targets were included to get CFS miRNA–FS mRNA pairs. Finally, the lncRNA–CFS miRNA pairs and CFS miRNA–FS mRNA pairs were imported and analyzed in Cytoscape 3.8.1 (Shannon et al., 2003) (https://cytoscape.org/). Pairs which could not form an lncRNA–miRNA–mRNA triplet were excluded. Alongside that, lncRNAs with a degree lower than three were also deleted. The ceRNA network was finally visualized by Cytoscape 3.8.1.

### Validation of the Expression Changes of FR lncRNA

C3H/10T1/2 cells were purchased from Procell Life Science and Technology Co., Ltd. and were then cultured at 37°C under 5% carbon dioxide using modified Eagle’s medium (MEM, Invitrogen) supplemented with 10% fetal bovine serum (FBS, Gibco), 100 U/ml penicillin, and 100 μg/ml streptomycin (Invitrogen). The Flexcell FX-5000TM Tension System (Flexcell International Corporation) was used to apply mechanical cyclical stretch. A total of 5 × 10^4^ cells were seeded onto the collagen type-I-coated BioFlex silicon plates per well for 24 h. Cells were then subjected to cyclical mechanical stimulation with 10% elongation, 0.5 Hz for 24 h. Control cells were cultured under the same conditions without stretch force. Once the programmed stimuli finished, the total RNA of cells was extracted by RNA-Quick Purification Kit (Esunbio, China). Then, 1 µg of RNA was transcribed to cDNA using a HiScript^®^ III RT SuperMix for qPCR (+gDNA wiper) (Vazyme, China) according to the manufacturer’s protocol. The qRT-PCR assay was carried out on an ABI QuantStudio 6 system using Taq Pro universal SYBR qPCR Master Mix (Vazyme, China) according to the manufacturer’s protocol. GAPDH was used as endogenous references. The 2^−ΔΔCt^ method was used to determine the relative quantification of gene expression levels. Primers for all the genes are listed in Table 1.

**TABLE 1:** Primer sequences of lncRNA validated by RT-qPCR.

LncRNA	Primer sequences (from 5′- 3′)
Gapdh	F: CAG TGC CAG CCT CGT CTC AT
	R: AGG GGC CAT CCA CAG TCT TC
Kcnq1ot1	F: AGT​GAG​CAC​GTT​CTG​TCT​GG
	R: ACA​GGT​AGG​TGC​CGT​AGT​CT
Snhg16	F: TGA​TGG​CAT​TGC​CTT​TTG​GC
	R: GCC​CAC​CAT​CTA​CCT​ATG​CC
Noard	F: GAC​CAC​AGC​CTT​TGT​TAG​GTG
	R: CCA​CCC​TAC​CAA​TCC​GTT​ATG​T
Snhg12	F: AAT​TCA​GGT​TTG​CAT​AGT​GGC
	R: CAT​GAC​CAG​TGT​CTG​TAC​TCA
Tug1	F: CTC​TGG​AGG​TGG​ACG​TTT​TGT
	R: GTG​AGT​CGT​GTC​TCT​CTT​TTC​TC

### Identifying the FS mRNA–FR lncRNA Interaction and Co-expression

Interaction relationships between FS mRNA and FR lncRNA were screened out based on the database of ENCORI, which recorded the pairing relationship supported by at least one experiment. Loop relationships were extracted from the ceRNA network and a Ce-loop network was constructed. For co-expression network construction, the expression profile of GSE112122 was first normalized to TPM (transcripts per kilobase million) value, and the Pearson correlation coefficient was then calculated among FS mRNAs and FR lncRNAs. A threshold was set as a COR of >0.8 and a *p*-value of <0.05.

### TF–LncRNA Regulation and Potential Feedback Circuit

Transcription activity is tightly regulated by TFs via their sequence-specific binding with the TFBSs on genome. We managed to map out the possible regulation of TFs on our FR lncRNAs for elucidation of the potential upstream mechanism. The prediction strategy was based on the position weight matrix (PWM) method (D’haeseleer, 2006). The upstream sequences of genes were downloaded from the University of California, Santa Cruz (UCSC) Genome Browser, and 1,000 bp were used for TFBS prediction of lncRNA upstream regulatory sites. The R 3.5.0 framework and TFBSTools package (Tan and Lenhard, 2016) (http://www.bioconductor.org/packages/release/bioc/html/TFBSTools.html) as well as JASPAR2016 package (Mathelier et al., 2016) (http://www.bioconductor.org/packages/release/data/experiment/html/JASPAR2016.html) were used for calculation, and the cutoff score was set as 95%. The TF–FR lncRNA regulation was later analyzed and visualized by Cytoscape 3.8.1. For possible bidirectional regulation between TFs and lncRNAs, we intersected the FR ceRNA as well as FTR ceRNA network with the TF list gained by TFBS prediction. TF–FR lncRNA pairs predicted to have a ceRNA relationship via miRNAs were then chosen. Triplets which could form the circle as TF–FR lncRNA–CFS miRNA–TF were finally included and visualized.

### Paper-Based Pathway Mapping of FR lncRNAs

Based on the extensively reported mechanosensitive pathways as well as those essential for bone development, we matched lncRNAs in our networks to possible mechanisms based on their known effects. Specifically, compelling masters of mechanotransduction such as YAP/TAZ, Wnt/*β*-catenin, BMP/Smad, and MAPK are the main focus. Possible mechanisms of lncRNAs were analyzed based on the reported research in bone and beyond.

### Identification of FS LncRNA and Pathway–LncRNA Network Construction

To corroborate our bioinformatics prediction, we reviewed the published articles concerning lncRNAs in bone cells undergoing simulated mechanical condition. Our searching strategy aimed to find research related to all kinds of mechanical condition, including tension, compression, fluid shear stress, vibration, microgravity, stiffness, topography, and so on. Besides, research irrelevant to the skeletal system was excluded. All data were updated to 2021/3/1. We named lncRNAs reported before as force-sensitive lncRNAs (FS lncRNAs). Furthermore, we built the pathway–lncRNA network integrating prediction results of FR lncRNA with verified data of FS lncRNA, which was then visualized by Cytoscape.

## Results

### Identification of FS and FTS mRNAs

**FIGURE 2:**
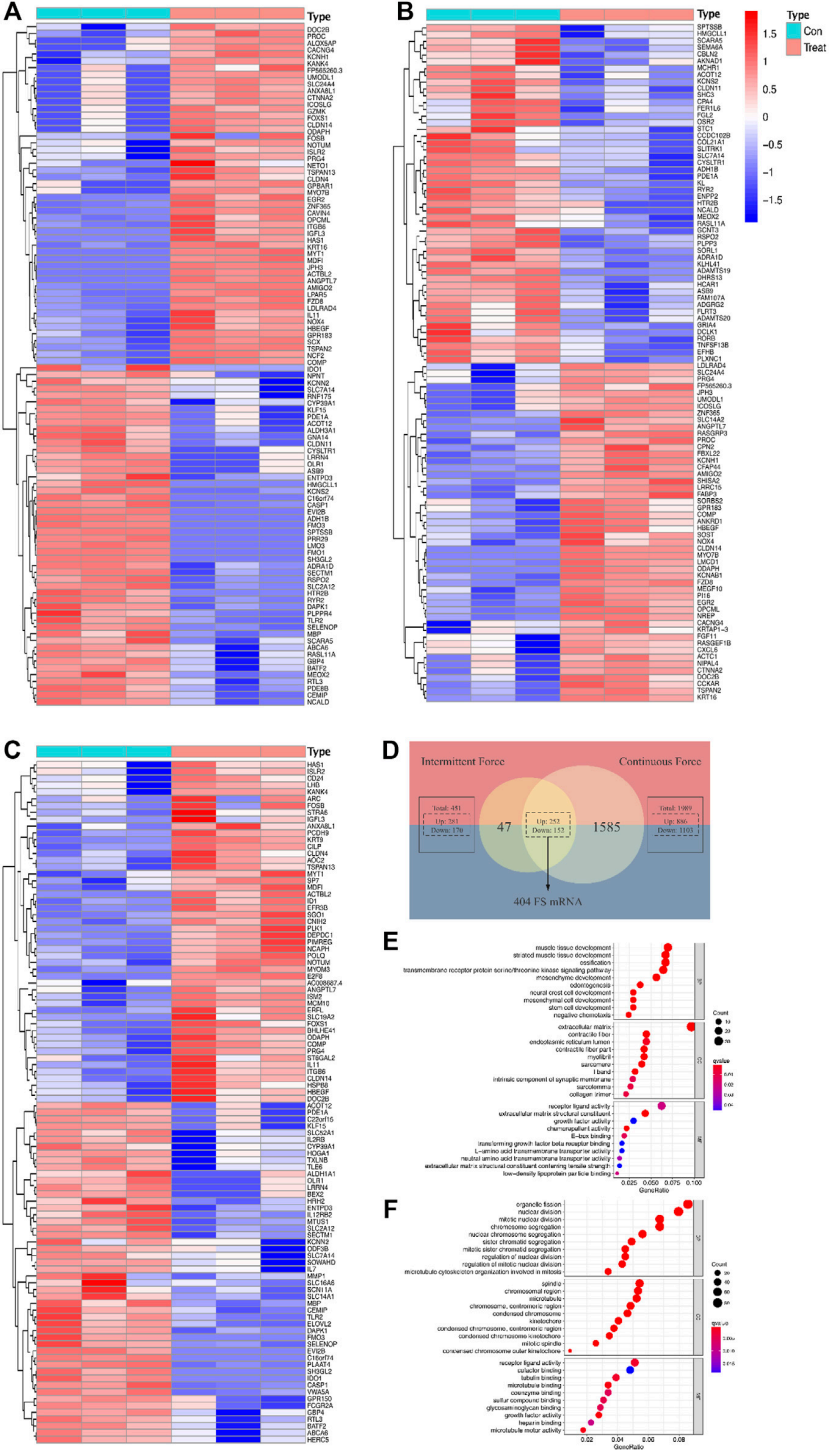
Differentially expressed profiles and functional analysis of mRNAs in a force-related way. Heatmaps display the top 50 genes with the highest values of |log2foldChange (FC)| in the intermittent force group **(A)**, static force group **(B)**, and different force type group **(C)**. Intersection of results gained from two force groups contributes to the list of force-sensitive mRNA (FS mRNA), which is visualized by the VENNY diagram **(D)**. Top 10 GO annotations grouped by BP, CC, and MF of FS mRNAs **(E)** and FTS mRNAs **(F)**. GO terms are ranked by the GeneRatio scores. The size and color of the dots represent the gene count and *q*-value score of the enrichment analysis, respectively.

As for the intermittent force group, 1,989 DE mRNAs were screened out, including 886 upregulated and 1,103 downregulated mRNAs. Additionally, 451 DE mRNAs were validated in the static force group, with 281 and 170 DE mRNAs found to be upregulated and downregulated, respectively. The discrepancy of the DE mRNA volumes between two force types conformed to the conclusion of the original study (Manokawinchoke et al., 2019). The intersection of DE mRNAs from intermittent and static force groups contributed to 404 genes in common, which were identified as FS mRNAs. Among them, 252 were promoted and 152 were inhibited after the mechanical loading regardless of types. Data analysis concerning force types contributed to 1,086 DE mRNAs, with 521 upregulated and 565 downregulated, which were defined as FTS mRNAs. Heatmap plots were used to assess differentially expressed genes, and a VENNY diagram was displayed for FS mRNA screening (Figures 2A–D).

GO annotation and enrichment analysis of the DE mRNAs in different groups provided us with a better understanding of the physiological process of PDLCs responding to mechanical force. Under the cutoff limitation of *p* < 0.05, 396 GO terms were screened out, with 359 in the biological process (BP), 21 in the cellular component (CC), and 13 in the molecular function (MF) based on the FS mRNAs. As for the FTS mRNA group, total terms of GO are counted as 582 with 535, 32, 15 for BP, CC, and MF, respectively. BP terms enriched in FS mRNAs are most related to development processes like cellular developments, bone mineralization, and ossification, verifying that the cellular differentiation processes were impacted by applying force. As for CC terms, extracellular matrix, contractile fibers, and communication elements are most enriched among FS mRNAs, suggesting that the force would significantly induce the reaction modes of cells. The mechanically responsive cellular activity could also be indicated by the MF terms enriched in stimulus reception and communication such as receptor ligand activity. Interestingly, when it comes to FTS mRNAs, the GO terms are largely connected with nuclear and chromosome activity, implying the possibility for transcriptional differences when reacting to different force types (Figures 2E,F).

### Validation of CFS miRNAs

**FIGURE 3:**
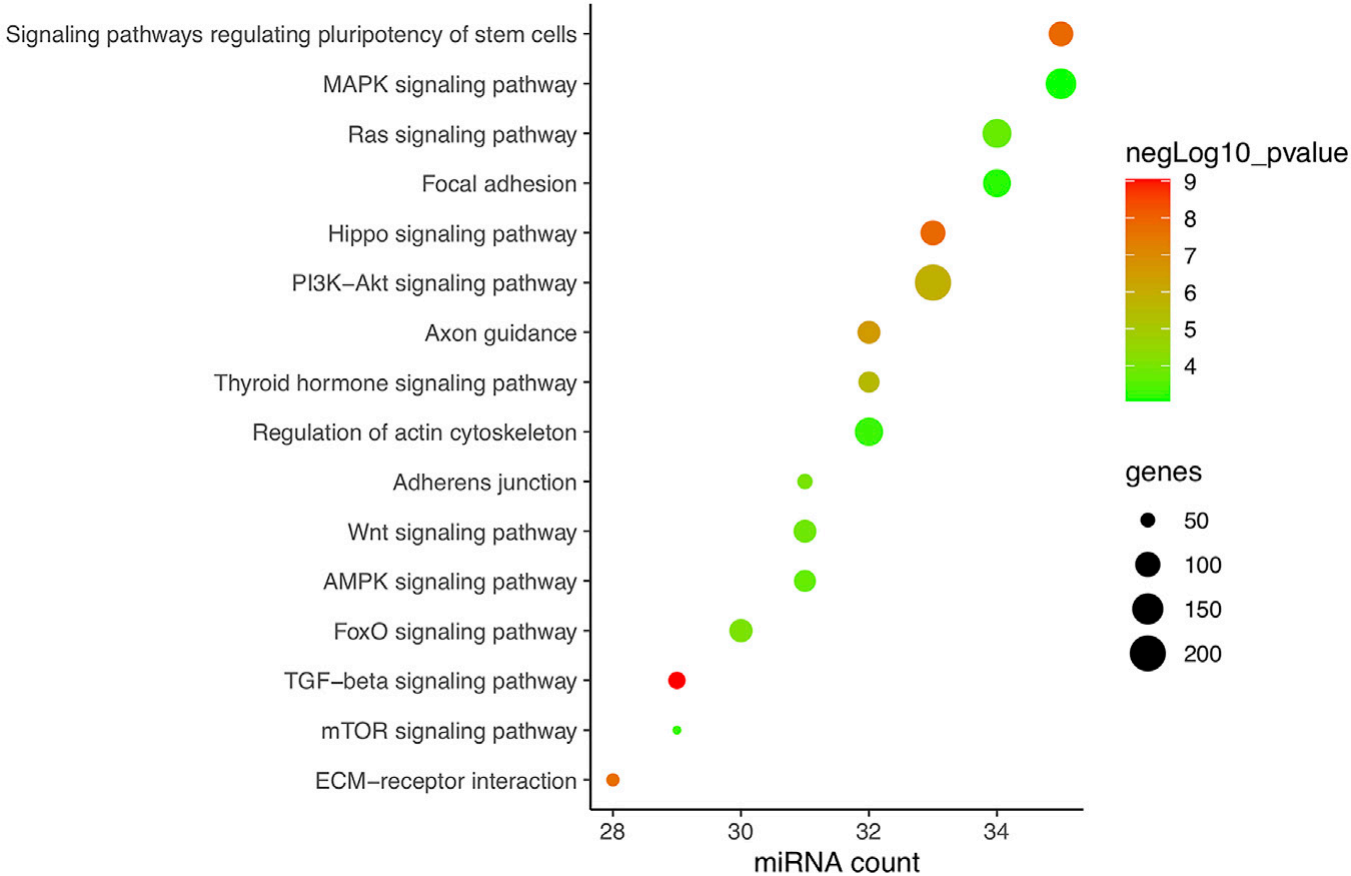
KEGG enrichment of CFS miRNAs corroborating the reliability of bioinformatics. Pathways are ranked according to *p*-value, and the top 22 are displayed after filtering those relating to cancer and chemical synthesis. KEGG terms included are highly consistent with verified FR pathways at present. Pathways excluded are proteoglycans in cancer, pathways in cancer, fatty acid biosynthesis, mucin-type *O*-glycan biosynthesis, morphine addiction, and melanoma (nos. 5, 6, 8, 11, 18, and 21, respectively).

In total, research carried out in MSCs, bone marrow stem cells (BMSCs), adipose-derived stem cells (ADSCs), PDLSCs, PDLCs, tendon-derived stem cells (TDSCs), alveolar bone cells (ABCs), primary rat osteoblasts (prOB), osteoblasts, MC3T3-E1 cells, MLO-Y4 osteocytes, and C2C12 cells was included and is listed in Table 2. Apart from hsa-miRNA, miRNAs that are conservative among species by sequence matching were also included. Finally, 38 CFS miRNAs, with 22 negatively and 16 positively related to osteogenic differentiation, were collected for further analysis. Based on CFS miRNAs, KEGG enrichment was carried out, and 59 pathways were screened out by mirPath. To focus on force/osteogenesis-related processes, we ranked pathways according to *p*-value and visualized the top 22 pathways after filtering those relating to cancer and chemical synthesis (Figure 3). Notably, TGF-*β*, Hippo, PI3K-Akt, Wnt, FoxO, and Ras signaling pathways are all recognized FS pathways, corroborating the credibility of the bioinformatics strategy. Furthermore, KEGG terms such as ECM–receptor interaction, adherens junction, regulation of actin cytoskeleton, and focal adhesion also ranked among the top 20, suggesting the involvement of ncRNA in controlling cell morphology to mechanical input. It is explainable for the cancerous pathway like proteoglycans in cancer, pathways in cancer, and melanoma (nos. 5, 6, and 21, respectively) to be enriched in our analysis, considering cancer represents the most common model in miRNA studies. Overall, KEGG results of CFS miRNAs highly conform to the established data, ensuring the confidence of the following analysis.

**TABLE 2:** Creditable force-sensitive miRNAs and related pathways.

Evidence		Samples	Force	miRNA	Osteo^a^	Target and pathways
Yuan et al. (2019)	function	prOB	CTS: 3% strain, 0.5 Hz, 4 h	miR-214	N	Pten, *β*-catenin, and ATF4
	qPCR			miR-30d-5p, miR-199a-3p	N	
	qPCR			miR-31-5p	P	
Wei et al. (2015)	function	hPDLSCs	CTS: 10% strain 1.0 Hz, 6, 12, 24, or 48 h	miR-21	P	ACVR2B (TGF-*β*)
Zuo et al. (2015)	function	FOB 1.19	CTS: 8% strain, 0.5 Hz, 72 h; MG: *in vivo* 28 days	miR-103a	N	Runx2
Guo et al. (2015)	qPCR	MC3T3-E1	CTS: 2,500 µε, 0.5 Hz, 8 h	miR-191*, miR-3070a	P	
	qPCR			miR-218, miR-33	N	
Zeng et al. (2019)	function	MLO-Y4	CTS: 2,500 με, 0.5 Hz, 8 h	miR-29b	N	Inhibited IGF-1 secretion
	qPCR			miR-713, miR-706, miR-703, miR-574-3p, miR-467b-3p, miR-466i/f-5p, miR-208a-3p	P	
	qPCR			miR-361-3p	N	
Liu et al. (2017)	function	rBMSCs	CTS: 10% strain, 1 Hz, 12 h	miR-503-5p	N	
	qPCR			miR-34c-3p, miR-326-5p	P	
	qPCR			miR-324-5p, miR-188-5p, miR-345-3p, miR-30a-5p, miR-29b-3p, miR-351-3p	N	
Wu et al. (2019b)	qPCR	hPDLC	CTS: 10% strain, 0.1 Hz, 24 h	miR-138-5p, miR-221-3p, miR-132-3p	P	
	qPCR			miR-133a-3p, miR-133a-5p, miR-210-3p	N	
Kanzaki et al. (2019)	function	hPDLC	CTS: 15% strain, 0.5 Hz, 24 h	miR-3198	N	Regulates OPG but not RANKL
			CF: 2.0 g/cm, 24 h			
Chen et al. (2015)	function	hPDLCs	CTS: 2% stretch, 0.1 Hz, 24 h	miR-29 family (a,b,c)	P	targeting at ECM genes: Col1a1 Col3a1 Col5a1
			CF: 2.0 g/cm, 24 h			
Iwawaki et al. (2015)	function	MC3T3-E1	CF: 2.0–4.0 g/cm^2^, 24 h	miR-494-3p	N	Fgfr2, Rock1
	qPCR			miR-146a-5p, miR-210-3p, miR-1247-3p	N	
Chang et al. (2017)	function	hPDLC	CTS: 12% strain, 0.1 Hz, 24, 48, or 72 h	miR-195-5p	N	WNT3A, FGF2, and BMPR1A
Chang et al. (2015)	qPCR	hPDLC	CTS: 12% strain, 0.1 Hz, 72 h	miR-195-5p, miR-424-5p, miR-1,297, miR-3607-5p, miR-145-5p, miR-4,328, miR-224-5p	N	
Luo et al. (2019)	function	hADSCs	CTS: 5% strain, 0.5 Hz, 2 h/day, 6 days	let-7i-3p	N	LEF1 (Wnt/β-catenin)
Wu et al. (2018)	function	hBMSCs	CTS: 10% strain, 0.5 Hz, 6 h/day, 7 days	miR-138	N	miR-138/PTK2(FAK-ERK1/2-Runx2)
Li et al. (2015)	function	mADSCs	CTS: 2000 με, 0.5 Hz, 2 h/day, 7 days	miR-154-5p	N	Wnt/PCP (RhoA/ROCK)
Mohan et al. (2015)	function	mOB	CTS: 2 Hz, 36 cycles/day, 2 weeks	miR-20a	P	IGF-I
Hu et al. (2016)	function	hBMSCs, hTDSCs, hADSCs	ESW: 0.16 mJ/mm^2^, 500 impulses	miR-138	N	miR-138-FAK-ERK1/2-RUNX2
Zhang et al. (2020)	function	MC3T3-E1	MG: 24, 48, 72 h	miR-30b/c/d/e	N	Runx2
Qin et al. (2019)	function	C2C12	MG: 12 h	miR-494	N	MYOD; BMPR-SMAD-RUNX2
	qPCR			miR-122a, miR-340	P	
Wang et al. (2016)	function	MC3T3-E1	MG: 48 h; FSS: 10 dyn/cm^2^, 1 h	miR-33-5p	P	Hmga2
Sun et al. (2015)	function	MC3T3-E1	MG: 48 h	miR-103	N	Cav1.2: calcium voltage-gated channel
Hu et al. (2015)	function	prOB	MG: 48 h	miR-132-3p	N	Ep300-Runx2
	qPCR			miR-139-3p, miR-339-3p	N	
	qPCR			miR-487b, miR-2,985, miR-34b	P	
Qi and Zhang, (2014)	function	hPDLC	FSS3, 6, 9, 12, 15 dyn/cm^2^, 6 h	miR-132	P	mTOR
Chen et al. (2016)	function	h/mPDLCs	OTM	miR-21	P	Pdcd4 (C-fos)

a Osteogenesis: N, negative; P, positive; CTS, cyclic tensile strain; CF, compressive force; MG, microgravity; OTM, orthodontic tooth movement; FSS, fluid shear strain; ESW, extracorporeal shockwave; rBMSCs/hBMSCs, rat/human bone marrow-derived stem cells; hADSCs, human adipose tissue-derived stem cells; h/mPDLSCs, human/mouse periodontal ligament stem cells; hPDLC, human periodontal ligament cells; m/prOB, mouse/primary rat osteoblast.

### FR CeRNA Network Visualization and FR LncRNA Exploration

**FIGURE 4:**
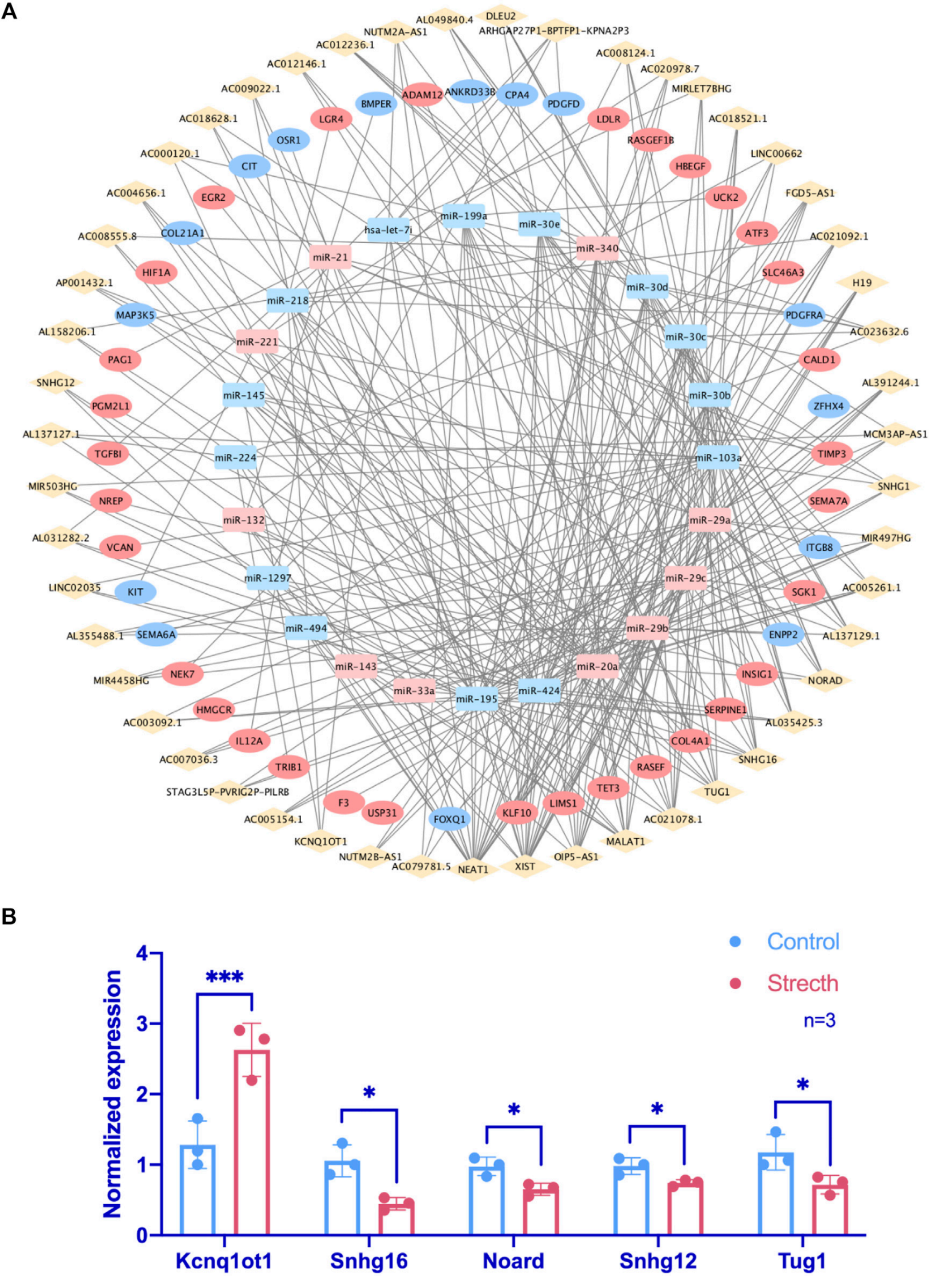
Identification of FR lncRNA through the ceRNA network and RT-qPCR validation. In the ceRNA network, rhombuses, squares, and circles stand for lncRNAs, miRNAs, and mRNAs, respectively. Upregulated RNAs are shown in pink, while downregulated ones are shown in blue. Gray lines represent regulatory relationships **(A)**. The validation of lncRNA expression changes via RT-qPCR under mechanical stretch was normalized to Gapdh (*n* = 3) **(B)**.

One hundred seventy-seven lncRNAs and 442 pairs of lncRNA–miRNA, as well as 1,445 target mRNAs and 2,463 miRNA–mRNA pairs, were predicted based on CFS miRNAs. The intersection of predicted mRNA targets and FS mRNAs was carried out, and 47 FS mRNAs and 79 mRNA-miRNA pairs were then included. Based on the 24 miRNAs identified by FS mRNA–CFS miRNA pairs, 147 lncRNAs and 408 CFS miRNA–lncRNA pairs were chosen for FR ceRNA network construction. Last, lncRNAs with a degree lower than three were excluded based on the network analysis carried out by Cytoscape. After filtration, 122 nodes as well as 340 edges were finally reserved, which include 51 lncRNAs, 24 miRNAs, and 47 mRNAs (Figure 4A). Strikingly, the network analysis displayed by Cytoscape indicated that all CFS miRNAs show a value of degree ≥5, and 14 out of the 24 CFS miRNAs have their degrees higher than 10, highlighting the core role as well as the credibility of our CFS miRNAs. Notably, the intricate and entwined edges around miR-20a, miR-195, and miR-424, the top three miRNAs with the highest degrees of 20, 29, and 31, respectively, suggest possible dominating roles of these miRNAs in mechanobiology, which is worthy of future exploration. As for lncRNA prediction, the exclusion of lncRNAs with a degree lower than three contributed to a more confidential outcome as 51 lncRNAs were finally included, named the FR lncRNAs. Interestingly, when ranked by degree, betweenness, or closeness, 13 lncRNAs, namely, NEAT1, XIST, OIP5-AS1, MALAT1, AC021078.1, TUG1, SNHG16, AC005261.1, AC021092.1, FGD5-AS1, H19, SNHG1, and AL035425.3, are always listed among the top 15 regardless of the indicator of ranking, highlighting their potential key roles in the mechanoresponsive process. For *in vitro* validation of our prediction, expression changes of 10 FR lncRNAs were checked by qPCR (Figure 4B).

### FR LncRNA Exploration Based on FS mRNA Interaction and Co-expression Network

**FIGURE 5:**
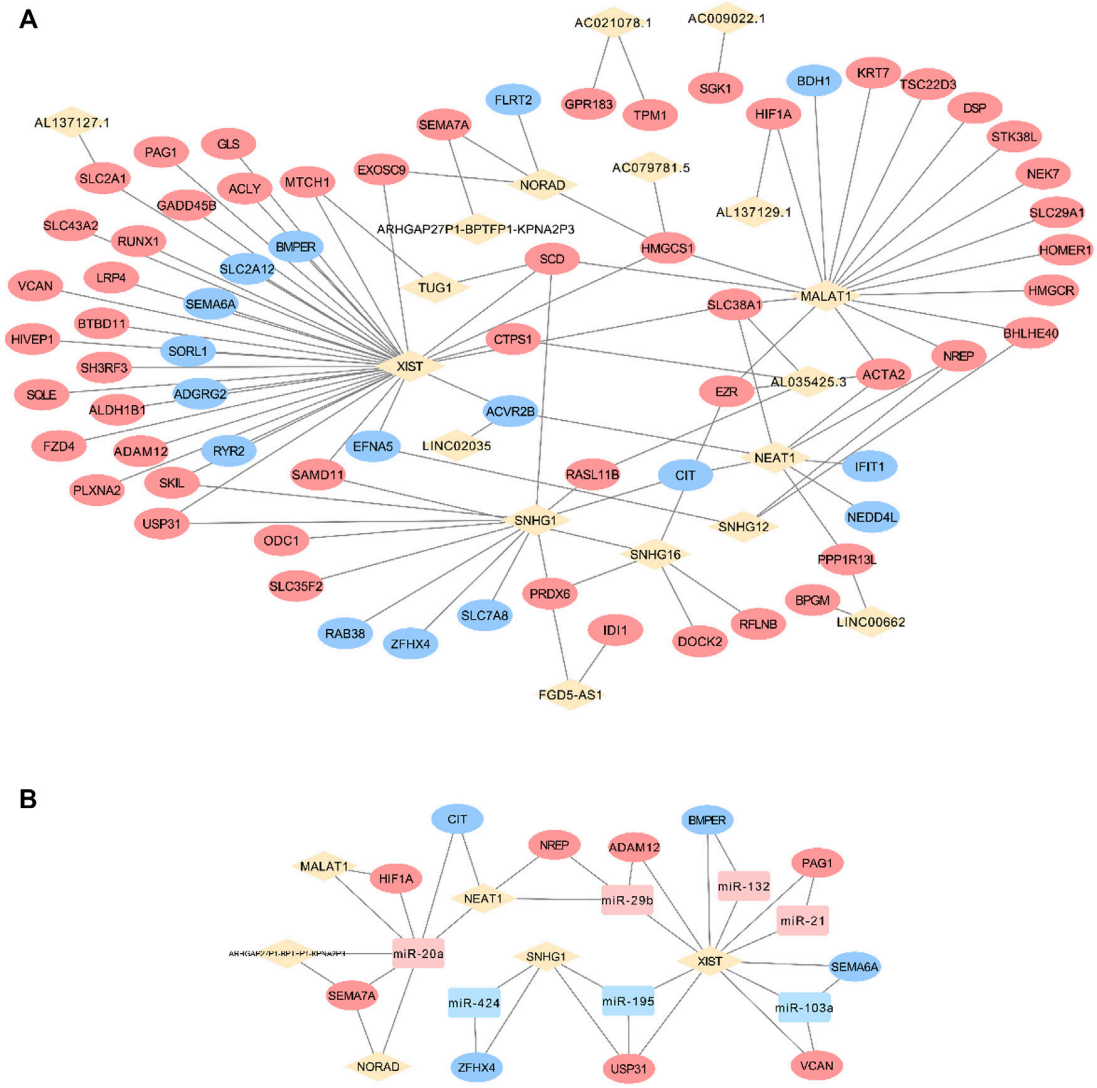
FS mRNA and FR lncRNA interaction. The direct interaction relationships were visualized **(A)** and the Ce-loop RNA network was also extracted **(B)**. Rhombuses, squares, and circles stand for lncRNAs, miRNAs, and mRNAs, respectively. Upregulated RNAs are shown in pink, while downregulated ones are shown in blue. Gray lines represent regulatory relationships.

**FIGURE 6:**
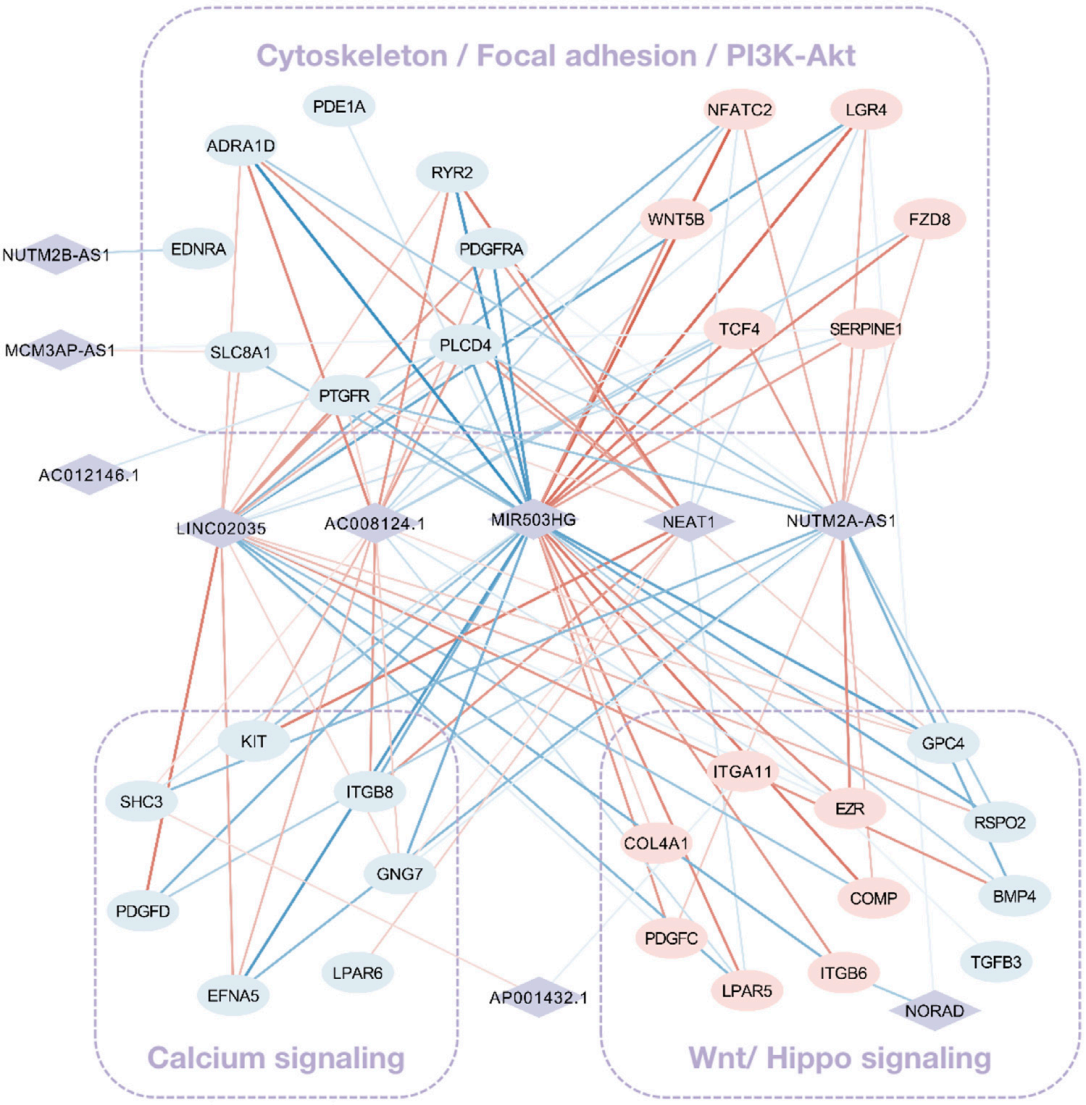
The co-expression network among FS mRNA and FR lncRNA. Rhombuses and squares stand for lncRNAs and mRNAs, respectively. Upregulated RNAs are shown in pink, while downregulated ones are shown in blue. Red and blue lines represent positive and negative correlations, respectively. The widths of lines are mapped to correlation scores.

Grounded on the list of FR lncRNAs gained from the strong evidence provided by FS mRNA–CFS miRNAs, the next goal is to explore the functionality of FR lncRNAs. To start with, lncRNA, as an intricate type, could not only impact mRNA indirectly though miRNA as a ceRNA mechanism, it could directly interact with mRNAs as well. Sixty-eight FS mRNAs and nine FR lncRNAs were verified to have direct interaction relationships. XIST, MALAT1, NORAD, and SNHG1/12/16 showed highly intertwined interaction with FS mRNAs (Figures 5A,B). Second, based on the guilt-by-association method, it is supposed that highly co-expressed genes during the biological process hold great possibility to share a similar biological function. Thus, novel lncRNAs’ function could largely be predicted by co-expressed protein-coding mRNAs (Liao et al., 2011). In our work, 910 pairs of FS mRNA–FR lncRNA showed Pearson’s correlation coefficients ≥0.8 (*p* < 0.05), which further verified the credibility of our prediction. For a BP-based explanation, we screened out several force-responsive pathways which were highly enriched among these pairs (*p* < 0.05), such as the PI3K-Akt signaling pathway, focal adhesion, regulation of actin cytoskeleton, calcium signaling pathway, and Wnt and Hippo signaling pathways. Connecting the co-expression network with a pathway could further suggest the potential roles of FR lncRNAs (Figure 6).

### Regulation of FR LncRNAs Realized by TFs and the Possible Bidirectional Interaction Between TF and LncRNAs

**FIGURE 7:**
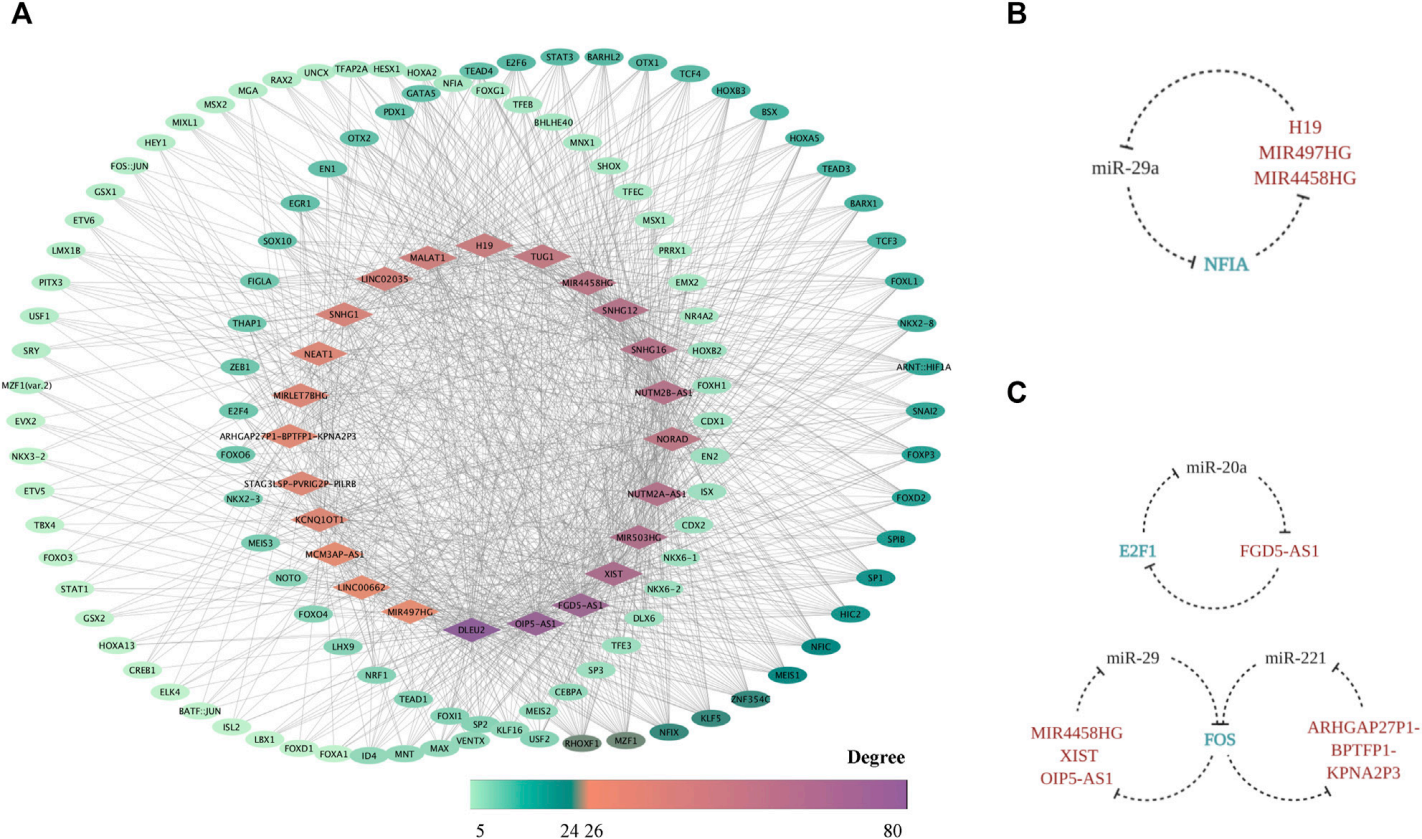
The FR TF–lncRNA regulatory network. The TF–FR lncRNA network is visualized with lncRNAs forming the middle circle while TFs with degree < 10 standing on the left and those with degree ≥ 10 on the right. Node colors are mapped to degree scores. **(A)** The feedback loops based on the triplet of TF (FS mRNA)–CFS miRNA–FR lncRNA **(B)** as well as the triplet of TF (FTS mRNA)–CFS miRNA–FR lncRNA **(C)** are displayed.

Based on the prediction through PWMs, 24 out of 51 FR lncRNAs were screened out, and 259 corresponding TFs were enrolled in the network, suggesting another potential mechanism of force-sensitive response. TFs with a degree lower than five were excluded for demonstration, and 121 TFs were finally reserved. RHOXF1, MZF1, ZNF354C, NFIX, KLF5, MEIS1, NFIC, HIC2, SP1, FOXD2, and FOXP3 are the top 11 TFs with a degree higher than 10, highlighting their key roles of potential regulation on lncRNAs. What bears our attention is that a large proportion of these TFs were explored to function in bone-related biological processes as well as mechanosensitive regulation, which could serve as validation for the plausibility of our network from one perspective. In turn, our work provided a systematic view and suggested the potential intricate pathways these TFs may involve in (Figure 7A) Interestingly, the shuttling of TFs from the cytoplasm to nuclear area provides the possibility for their bidirectional interaction with lncRNAs. From one perspective, as the mRNA target of miRNA sponged by lncRNAs in the cytoplasm, TFs are exposed to the epigenetic modification by lncRNAs. However, after TFs translocated to the nuclear area, it is the TFs which bind to the upstream transcripts of lncRNAs that master the transcription regulation. Hence, we explored the possibility of a triplet circular network in both the FS mRNA group and FTS group. Fortunately, we found NFIA-(MIR4458HG/H19/MIR497HG)-miR-29a-NFIA based on the FS group (Figure 7B), and the regulation effect between miR-29 and NFIA in bone remodeling was already confirmed in a previous study (Franceschetti et al., 2013). Meanwhile, the regulation loops were also identified in the FTS group as FOS-(ARHGAP27P1-BPTFP1-KPNA2P3)-miR-221-FOS, FOS-(MIR4458HG/XIST/OIP5-AS1)-miR-29a/b/c-FOS, and E2F1-(FGD5-AS1)-miR-20a-E2F1 (Figure 7C). In conclusion, NFIA, FOS, and E2F1 showed significant alternation of expression profiles when exposed to the force-related environment while embodying the potential to form the force-sensitive circuit with lncRNAs and miRNAs, which could be studied in the future.

### Elucidation of the Downstream Pathway Map of FR lncRNAs

Literature retrieval provided evidences of lncRNAs’ effects on mediating the cellular response to mechanical cues. Here, we mapped out a systematic evaluation of the pathways that our FR lncRNAs may involve in (Table 3). NEAT1 and H19, with high scores in our network, have gained wide recognition in mechanobiology by previous study, which confirmed the reliability of our work. Remarkably, studies of NEAT1 or H19 were carried out under different force conditions in various cells, which indicated that these lncRNAs may be universal mechanoresponsive factors like our study aimed to define (Hirose et al., 2014; Heo et al., 2015; Le et al., 2016; Li et al., 2017, 2018; Izadpanahi et al., 2018; Wu et al., 2018; An et al., 2019; Wang S. et al., 2019; Zhang Y. et al., 2019; Huang et al., 2019; Todorovski et al., 2020).

**TABLE 3:** Pathway mapping of force-related lncRNAs.

LncRNA	Clarified information		
	Background	Reported target and function	Pathway
NEAT1	HBMSC (Zhang X et al., 2019)	Promoting osteogenic differentiation via miR-29b-3p/BMP1 axis	BMP
	cancer cell lines (Todorovski et al., 2020)	Mediate the mechanomemory to substrate stiffness via H3K27me3 activity	EZH2
H19	HBMSC (Wu et al., 2018)	Promoting osteogenesis under tension via sponging miR-138 to release PTK2	FAK-ERK1/2-Runx2
	UMR-106 (Li et al., 2018)	Positively related to osteogenesis under mechanical unloading *via* DNMT1/H19/Erk	ERK-MAPK
	UMR-106 (Li et al., 2017)	Positively related to osteogenesis under mechanical unloading *via* Dkk4	Wnt/*β*-catenin
	MC3T3-E1 (Wu et al., 2019a)	Promoting matrix mineralization via miR-185-5p/IGF1	IGF1
	HBMSCs (Bi et al., 2020)	Promoting osteogenic differentiation *via* miR-140-5p/SATB2	SATB2
	REMSCs (Gong et al., 2018)	Promoting osteogenic differentiation *via* miR-22, miR-141/*β*-catenin	Wnt/*β*-catenin
	HBMSC (Xiaoling et al., 2020)	Elevates cell proliferation and differentiation of BMSCs *via* miR-19b-3p	
KCNQ1OT1	HBMSCs (Wang J. L et al.,2020)	Promoting osteoblast generation *via* miR-320a/Smad5	Cbfa1/Runx2
	HBMSCs (WangC.-G. et al., 2019)	Promoting osteogenic differentiation *via* miR-214/BMP2	BMP2
	MTSPCs (Yu et al., 2018b)	Promoting osteogenic differentiation *via* miR-138	RUNX2/PPARγ
	Hc-a cells (Gu H. et al., 2019)	A potential biomarker of delayed fracture healing of patients promoting cellular proliferation and inhibiting apoptosis	Wnt/*β*-catenin
TUG1	MTSPCs (Yu et al., 2020)	Promoting osteogenic differentiation via promoting the ubiquitination of bFGF	bFGF
	HPDLSCs (He et al., 2018)	Promoting osteogenic differentiation *via* lin-28 homolog A (Lin28A)	
	HPDLSCs (Wu D. et al., 2020)	Promoting osteogenic differentiation *via* miR-222-3p/Smad2/7	Smad
	Osteoblast (Liu S. C et al., 2019b)	Promoting osteoblast proliferation and differentiation	Wnt/*β*-catenin
	Valve interstitial cells (Yu C. et al., 2018)	Promoting osteogenic differentiation *via* miR-204/Runx2	Runx2
SNHG1	HBMSCs (Xiang et al., 2020)	Attenuating the osteogenesis *via* the miR-101/DKK1 axis	Wnt/*β*-catenin
	MBMSCs (Jiang et al., 2019)	Inhibiting osteogenic differentiation *via* Nedd4	p38 MAPK
	Prostate cancer (Chen et al., 2020)	Binding to EZH2 and exerting proto-oncogene effect	Wnt/*β*-catenin, PI3K/AKT/mTOR
	Osteosarcoma cells (Deng et al., 2019)	A negative regulator via miR-101-3p/ROCK1 pathway	ROCK1
MALAT1	RBMSCs (Zheng et al., 2019b)	Inhibiting osteogenic differentiation	MAPK
	Esophageal cancer (Yao et al., 2019)	Direct binding to enhance YAP activity	YAP/TAZ
	Acute pancreatitis (Gu L. et al., 2019)	Forming a loop as MALAT1/miR-194/YAP1	YAP/TAZ
	Non-small-cell lung cancer (Jin et al., 2019)	Forming a loop as MALAT1/miR-1914-3p/YAP	YAP/TAZ
	Valve interstitial cells (Xiao et al., 2017)	Promoting osteogenic differentiation *via* miR-204/Smad4	Smad
	Osteosarcoma (Cai et al.,2016)	Facilitating the metastasis	RhoA/ROCK
NORAD	Lung/breast cancer (Tan et al.,2019)	Transcriptionally repressed by the YAP/TAZ-TEAD complex	YAP/TAZ
	Hepatocellular carcinoma (Kawasaki et al.,^2018^; Yang et al.,2019)	Regulating epithelial-to-mesenchymal transition-like phenotype	TGF-*β*
		Promoting cancerous progression *via* miR-202-5p	
	Breast cancer (Zhou et al.,2019)	Promoting cancerous progression	TGF-β
OIP5-AS1	Valve interstitial cells (Zhu et al., 2019)	Promoting osteogenic differentiation via miR-137/TWIST11	TWIST11
SNHG16	Colorectal cancer (Christensen et al.,2016)	Regulated by Wnt activity	Wnt/*β*-catenin
	Cervical cancer (Wu W. et al., 2020)	Function via the SNHG16/miR-128 axis	Wnt/*β*-catenin
LINC-00662	Hepatocellular carcinoma (Tian et al., 2020)	Facilitating WNT3A secretion	Wnt/*β*-catenin
MCM3AP-AS1	Chondrocytes (Gao et al.,2019)	Increasing apoptotic rate of chondrocytes via miR-142-3p/HMGB1

Though research of lncRNAs’ involvement in mechanotransduction of the bone remains relatively void, studies of other cellular systems exploring essential pathways of force sensing could also fuel our understanding. As aforementioned, once the mechanical cues are transformed as intracellular signals, several intersected pathways are then involved to conduct the following cytoplasm-to-nuclear information delivery. RhoA/ROCK, Hippo/LATS1/2 kinase cascade, YAP/TAZ, AKT, JUN/p38 MAPK, BMP/Smad1/5/8, TGF-*β*/Smad2/3, and canonical and noncanonical Wnt pathways are all crucial factors involved in mechanobiology (Azzolin et al., 2012; Teramura et al., 2012; Furumatsu et al., 2013; Ji et al., 2014; Zhang et al., 2015; Gao et al., 2016; Panciera et al., 2017; Stanley et al., 2019). Therefore, lncRNAs predicted in our network, connecting these crucial factors and pathways, hold the promise to be key players in the process and are listed in Table 3, which could support our prediction as well boost downstream research.

Nevertheless, the project of lncRNA in the mechanical field is far from complete. For a deeper understanding, we also explored the possible mechanoresponsive and osteogenic pathways that these lncRNAs may participate in. H19, KCNQ1OT1, TUG1, SNHG1, and MALAT1, highlighted in our network, are closely correlated with osteogenesis via the ceRNA mechanism and beyond, which mainly involves BMP/Smad, RUNX2, MAPK, and Wnt/*β*-catenin signaling pathways (Yu C. et al., 2018; Gong et al., 2018; He et al., 2018; Gu H. et al., 2019; Liu H. et al., 2019; Wang C.-G. et al., 2019; Wu et al., 2019a; Jiang et al., 2019; Wu D. et al., 2020; Bi et al., 2020; Wang J. L et al., 2020; Xiang et al., 2020; Xiaoling et al., 2020; Yu et al., 2020), and detailed relationships are concluded in Table 4.

**TABLE 4:** Creditable force-sensitive lncRNAs and related pathways.

Evidence		Samples	Force	lncRNA	Exp	Pathway
Liu H. et al. (2019)	HiSeq 2000 (Illumina)	OCCM-30	CF	70 lncRNAs: 57 upregulated, 13 downregulated		KEGG pathway analyses of DEGs: HIF-1, FoxO, mTOR, Notch, and Rap1 signaling pathways
	qPCR			Prkcz2, Hklos, Trp53cor1, Gdap10, Ak312-ps	Up	
Hu et al. (2017)	Affymetrix GeneChip	MC3T3-E1	RWVB	857 lncRNAs: 168 upregulated, 689 downregulated		
	Bioinformatics qPCR			NONMMUT044983	down	Ptbp2: CaV1.2 calcium channel transcript
				NONMMUT018832		Tnpo1: nuclear translocation of oxytocin receptors
				NONMMUT023474		Ext1: BMP signaling
Huang et al. (2019)	HiSeq 2000 system (Illumina)	hPDLSCs	CF	90 lncRNAs: 72 upregulated, 18 downregulated		KEGG of DEGs: ECM–receptor interaction, focal adhesion, HIF-1, PI3K/Akt, protein digestion and absorption, and glycolysis/gluconeogenesis
	qPCR							
				FER1L4, HIF1A-AS2, MIAT, NEAT1, ADAMTS9-AS2, LUCAT1	up	
				MIR31HG, DHFRP1	down	
Huang et al. (2021)	functional	PDLSCs; Mice	CF OTM	Fer1l4	up	AKT/FOXO3
Zhang X. et al. (2019)	functional	HPDLCs; Rats	CF OTM	DANCR	up	miR-34a-5p/DANCR/Jagged1 (Notch signaling pathway)
Li et al. (2017)	Illumina HiSeq 2500	Rats	HLU	464 lncRNA: 83 upregulated, 381 downregulated	
	bioinformatics			H19	down	Dkk4/Wnt/β-catenin signaling; TGF-beta, and tight junction pathways
	functional					
Wu et al. (2018)	functional	hBMSCs	CTS	H19	up	H19–miR-138–PTK2(FAK)
Yan et al. (2021)	functional	U-MSCs to chondrocyte; rats	RCCS	H19	up	RCCS significantly promoted exosome production and exosomal lncRNA H19 at 36 rpm/min within 196 h
Li et al. (2018)	functional	UMR-106; rats	HLU	H19	down	DNMT1–hypermethylation of H19 promoter–ERK signaling; TGF-β, WNT, and JAK-STAT pathways
Zhang et al. (2018)	Affymetrix GeneChip	C2C12; Mice	HLU	lncMUMA	down	miR-762/MyoD
			RPM			
Wang M et al. (2020)	Microarray functional	hBMSCs	Topography	lncRNA PWRN1-209	up	integrin-FAK-ALP signaling
Wang Y et al. (2020)	functional	MC3T3-E1; Mice	HLU	ODSM	up	partially dependent on miR-139-3p/ELK1
Liu et al. (2016)	Microarray (Arraystar)	human chondrocytes	CTS	107 lncRNAs: 51 upregulated, 56 downregulated
	functional			lncRNA-MSR	up	miRNA-152/TMSB4
	microarray			lncRNA-CIR	up	vimentin
Izadpanahi et al. (2018)	bioinformatics	hASCs	Topography	MEG3	up	controlled by miR-125b
	qPCR					
	qPCR			H19	up	BMP signaling
Wang K et al. (2020)	functional	MC3T3-E1	Clinostat	OGRU	down	miR-320-3p/Hoxa10 axis-Runx2
		Mice				

CF, compressive force; RWVB, rotating wall vessel bioreactor; OTM, orthodontic tooth movement; HLU, hind limb unloading; CTS, cyclic tensile strain; RCCS, rotary cell culture system; RPM, random positioning machine; hPDLSCs, human/mouse periodontal ligament stem cells; hPDLC, human periodontal ligament cells; hBMSC, human bone marrow-derived stem cells; hADSCs, human adipose tissue-derived stem cells hADSCs, human adipose tissue-derived stem cells.

### Pathway–LncRNA Network Building Combining Predicted and Established Data

Genome-wide sequencing and bioinformatics analyses, as potent tools in ncRNA research, facilitate an overall understanding of lncRNA and were utilized by researchers to target ideal genes and molecules. To begin with, we conclude sequencing outcomes and computational strategies concerning FS lncRNAs published in recent years in Table 4, which could, on one hand, provide hints to future researches and, on the other hand, attenuate the improving quality and popularity of bioinformatics analysis.

**FIGURE 8:**
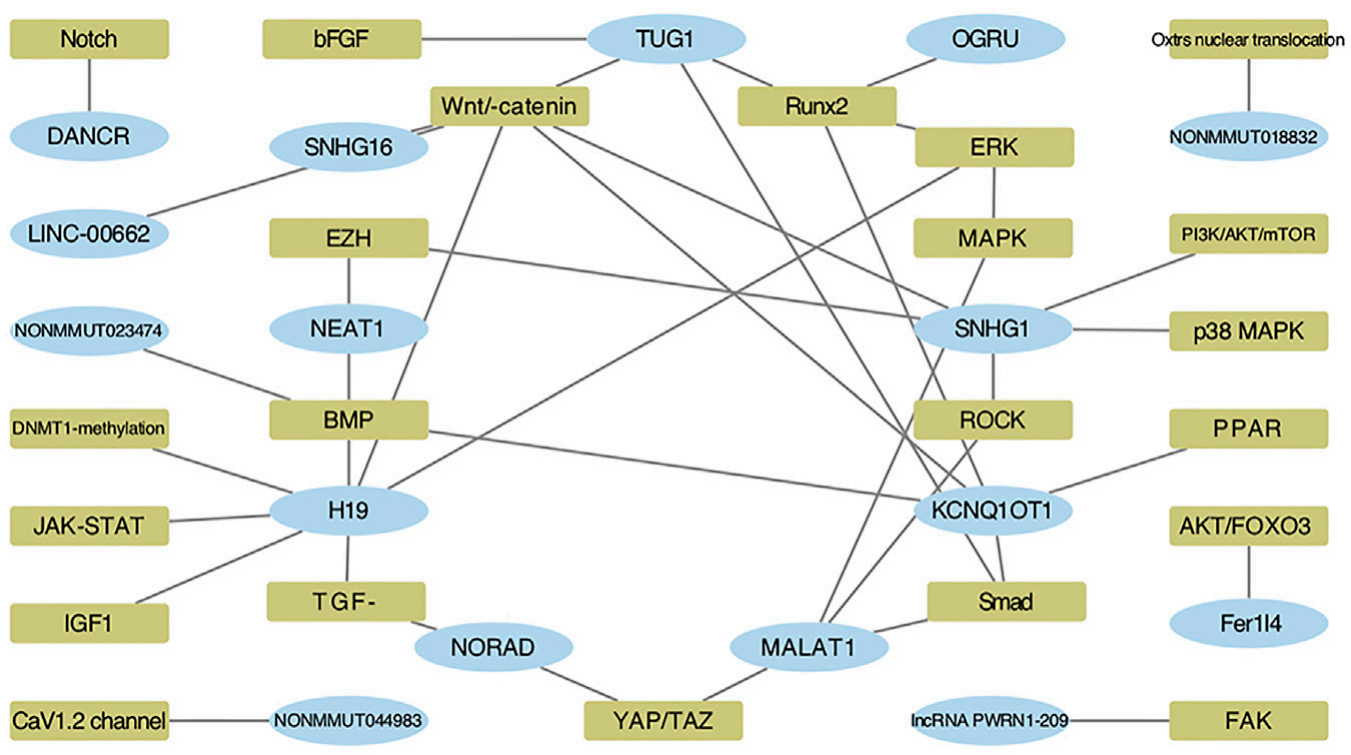
Intricate pathway network provoked by mechanical input is modulated by lncRNAs. Blue ellipses and green squares stand for FR lncRNAs and verified pathways, respectively.

Based on these published articles, we integrated the predicted pathways detailed in the last section with the proven ones, to illustrate the intricate pathway network of osteogenesis provoked by mechanical input modulated by lncRNAs (Figure 8). An overview of the pathways could stand as a valid proof of the conformity between predicted data with confirmed ones. More importantly, a pathway-centered network could provide insightful views of the interaction between lncRNAs. For instance, the Wnt/*β*-catenin signaling pathway displays a connection with lncRNAs as H19, SNHG1, SNHG16, TUG1, KCNQ1OT1, and LINC-00662, suggesting the possible interactive relationships of ncRNA modulation. The same goes for pathways like YAP/TAZ, BMP, Smad, and TGF-*β*. Though the function mode of ncRNAs as working via the network has long been recognized, previous studies mostly concentrated on the connections predicted by molecular bonding. Our work introduced a novel strategy to explore the interaction though pathway connection, which could be more specific when applying to a set of biological process like mechanical stimulation.

**FIGURE 9:**
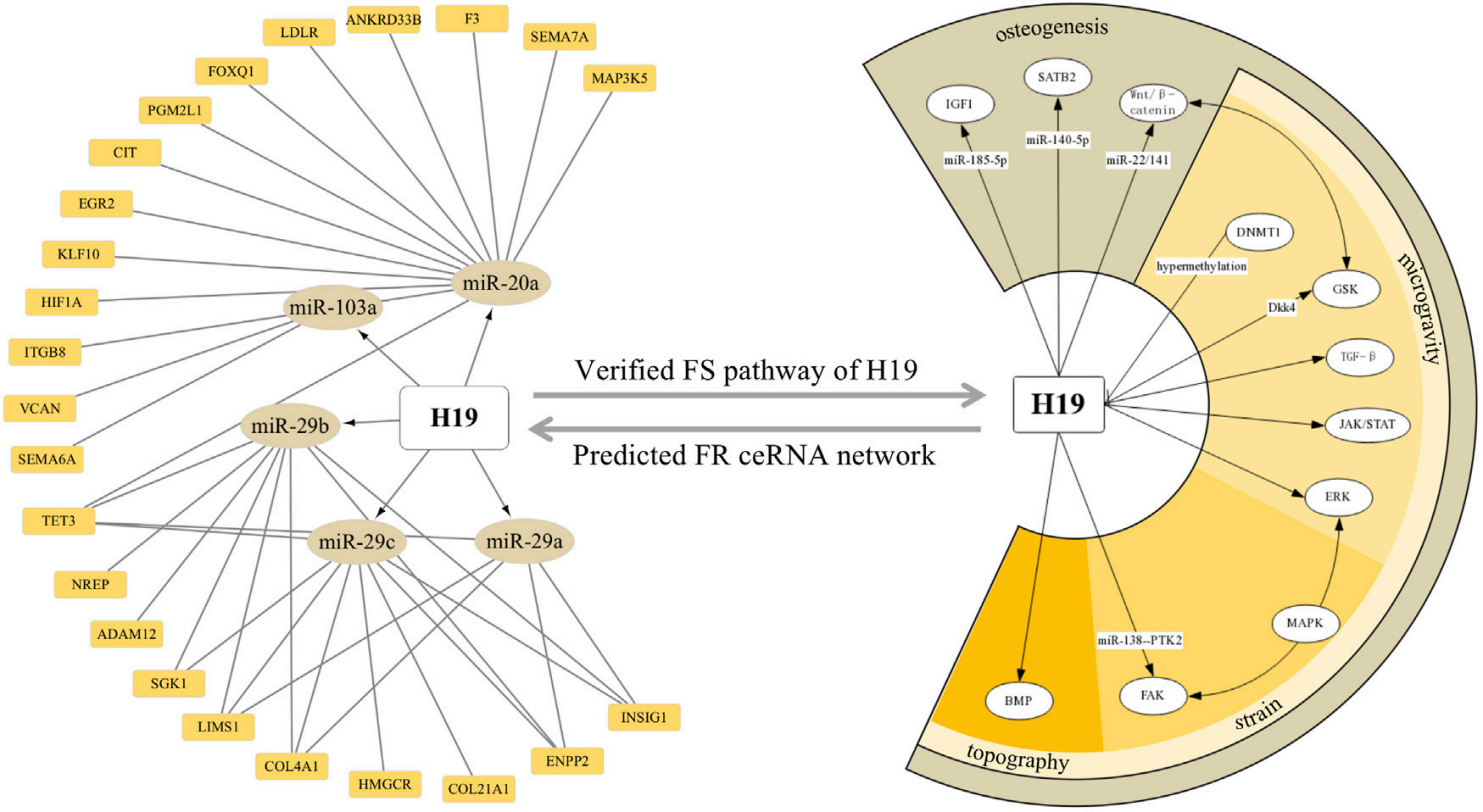
ceRNA network and downstream pathways revealed H19 as a key FS lncRNA confronting the mechanical input. Orange squares and dark yellow ellipses in the left semicircle stands for FS mRNA and CFS miRNA related to H19, respectively. White ellipses in the right semicircle represent the verified pathway H19 involved in FS condition and osteogenic processes.

Moreover, a combinative strategy based on our prediction and verified background would also provide new insights into a particular molecule. For instance, considering the centric role of H19 in force response, revealed by both our network analysis and established data, we integrated and visualized the ceRNA modulation and pathways H19 stimulates by confronting the mechanical input (Figure 9). Certificated roles of H19 could echo our prediction, while its complex interaction with CFS–miRNA and FS mRNA would suggest a promising field of research.

## Discussion

Mechanical force, which used to be so prevailing that it was often taken for granted, is now gaining the spotlight as a crucial factor for homeostasis maintenance (Wang et al., 2018). Particularly, as the supporting organ receiving and confronting complex force stimuli, the bone represents one of the most active tissue undergoing frequent remodeling, responding to mechanic cues (Yong et al., 2020). Outstandingly, though ncRNAs largely outnumber coding ones and master various properties, lncRNAs, as one type of ncRNA verified to have a diversified function mode and conformation, are less explored for their function in a mechanosensitive way. Aiming at providing a glimpse into universal lncRNA responding to mechanical stress, we first constructed a ceRNA network based on FS mRNAs and CFS miRNAs, which were individually screened out from the intersection of DE mRNA under different mechanical environments and from paper-based experimental data of high confidence. Consequently, lncRNAs involved in our ceRNA network with high degree were identified as FR lncRNAs. We carried out the functional analysis of FR lncRNAs from three aspects, namely, the FS mRNA interaction, TF regulation, and pathway network. To start, the direct interactions among FS mRNA and FR lncRNAs were displayed as well as the co-expression networks. Second, we explored the potential upstream regulation mediated by TFs on lncRNAs and put forward the underlying crosstalk loop between them, demonstrating the possible regulatory transcriptional activities. As for the downstream exploration, pathways concerning osteogenesis and mechanotransduction were mainly focused for the overview of FR lncRNAs. Later on, we developed combinative strategy to illustrate our predicted data against established ones of FS lncRNA, constructing an lncRNA-pathway network and identifying H19 as a critical factor in mechanobiology with more potential roles. In conclusion, our work contributed to a systematic prediction of a quartet network with lncRNAs at its core, elucidating the list of potential FR lncRNAs as well as their functional modes based on creditable evidence, suggesting a promising field worthy of further exploration and a new mode of bioinformatic analysis combining sequencing data, paper-based evidence, and computational prediction.

As the core component of our analysis, miRNAs and lncRNAs are no more than the so-called “noise” of transcriptome. Speculation as well as evidences are growing that ncRNAs may function in a more extensive way in the bone and beyond (Chen et al., 2017; Fang et al., 2019; Zhu et al., 2019). The past few years have seen the topic of miRNA’s role in the mechanical condition rush into the forefront (Chen et al., 2017; Wang et al., 2018; Fang et al., 2019), which was also concluded in our work. The FR ceRNA network demonstrated that miR-195, miR-424, and miR-20a are the top three miRNAs with the highest degree. Coherently, presented studies confirmed that miR-195 plays as an inhibitor of PDLC osteogenesis in both mice and human, targeting essential bone formation genes such as WNT family member 3A (WNT3A), fibroblast growth factor 2 (FGF2), and bone morphogenetic protein receptor-1A (BMPR1A) (Chang et al., 2017). Meanwhile, miR-424 was identified as one of the core miRNAs of tension-force-induced bone formation of PDLC in a former study (Chang et al., 2015). Additionally, *in vivo* study based on loaded and unloaded tibia of mice indicated that miR-20a was the only member of the miR17/92 cluster, which displayed significant changes responding to force (Mohan et al., 2015). Alongside these reported functions, our network indicated a more entwined relationship of these three miRNAs with mechanotransduction, which is open for further exploration. What also merits our attention are both the consistency and the discrepancy displayed by miRNAs under different stimuli or in diversified cellular environments. For instance, miR-33-5p promotes osteoblast differentiation regardless of the types of mechanical cues as MG or fluid shear stress (Wang et al., 2016). MiR-494 is a negative regulator of osteogenesis under both compressive and unloading conditions in MC3T3-E1 cells, C2C12 cells, and osteoblasts (Iwawaki et al., 2015; Qin et al., 2019). In hPDLCs, miR-29b shows altered expression level to both cyclic stretch and compression forces (Chen et al., 2015). However, it is only responsive to the mechanical tensile strain in osteocytes, but not osteoblasts of mouse (Zeng et al., 2019). MiR-138 was proven to damper the osteogenesis of MSCs regardless of their origins as bone, adipose, or tendon under the extracorporeal shockwave or cyclic strain (Hu et al., 2016; Wu et al., 2018). However, Yun Wu et al. (2019b) found miR-138-5p to be upregulated in qPCR and high sequencing under the cyclic strain of hPDLCs, which lacks reasonable explanation for the difference. Generally, though deeper exploration and validation should be carried out for the specific variance, the mechanosensitive properties of miRNAs remain coherent among cells and forces, supporting our systematic evaluation of the force-sensitive network.

Notably, another crucial prediction network in our study is carried out to evaluate the TF–lncRNA pairs, which was highlighted as an essential regulator under mechanical cues and beyond (Pylayeva-Gupta, 2011; Alam et al., 2014; Ji et al., 2020). Strikingly, TFs predicted to be the regulators of FR lncRNAs in our network are grounded for their roles in mechanotransduction and bone formation. Among the top 11 TFs with the highest degree, KLF5 mediates the response of hPDLCs to cyclic tensile stress and contributes to the progenitor maintenance in bone marrow via Rab5-β1/β2 integrin trafficking (Taniguchi Ishikawa et al., 2013; Gao J. Y. et al., 2018). Sensing the tensile forces, SP1 would enhance its interaction with p38 with elevated phosphorylation and activate its regulation on filamin-A (D’Addario et al., 2002). One orthodontic tooth movement model also proposed a mechanotransduction mode involving FAK, Ras-GTPase, and ERK1/2 regulating ephrin B2 via SP1 in periodontal ligament fibroblasts (PDLFs) (Diercke et al., 2011). The LINC00511/miR-150-5p/SP1 feedback loop in osteoarthritis (OA) was proven to master the extracellular matrix synthesis of chondrocyte (Zhang L. et al., 2020). Meanwhile, SP1 and MZF1 contribute to, whereas SP3 antagonizes, N-cadherin promoter activity in osteoblasts (Le Mée et al., 2005). MZF1, on the other hand, would regulate the bone formation of hMSCs via the osteopontin–MZF1–TGF-*β*1 linked pathway (Driver et al., 2015). NFIC was valued as a transcriptional switch of BMSCs to osteogenic or adipogenic differentiation for its interaction with diversified factors such as TGFβ1, KLF4, Wnt/*β*-catenin, and Runx2 (Lee et al., 2014; Roh and Park, 2017; Zhou et al., 2017; Huang et al., 2020). NFIX, regulated by miR-25-3p, also turned out to be a positive regulator of osteoclast proliferation and bone ossification (Driller et al., 2007). Elucidating TFs and their interactive relationship with FR lncRNAs, on one hand, contributes to a better understanding of the regulation network in mechanical transduction and, on the other hand, corroborates our prediction work as a qualified one worthy of further study.

As the main part of our studies, FR lncRNAs also show coincidence with a previous study. Remarkably, NEAT1, as the FR lncRNA with the highest score in our network, may exert an extensive epigenetic role in mechanotransduction. Elevated expression of NEAT1 in PDLSCs confronting compressive force (CF) was verified (Huang et al., 2019), and it was proven to connect the mechanosensitive epigenetic silencing *via* PRC2-H3K27me3 (Hirose et al., 2014; Le et al., 2016; An et al., 2019; Todorovski et al., 2020). In the myogenic process of C2C12 cells, this role was suggested to depend on the interaction between NEAT1 and EZH2 (Wang S. et al., 2019). Meanwhile, in PDLSCs, EZH2 is responsible for the global change of H3K27me3 and H3K27ac activity to encode the mechanical information onto chromatin when CF was applied both *in vivo* and *in vitro* (Heo et al., 2015). Thus, NEAT1 holds the promise of being one epigenetic modification strategy utilized by cells to memorize the mechanical cues after the withdrawal of physical stimuli. Considering its verified osteogenic promotion role in hBMSCs (Zhang Y. et al., 2019), a deeper investigation of NEAT1 functionality in connecting force and bone remodeling is worth exploring. H19, another lncRNA involved in our network, can also be a case in point. Under mechanical tension, H19 was elevated as a miRNA sponge of miR-138 to release PTK2, which codes FAK, inducing osteogenesis of hBMMSC via the FAK-ERK1/2-Runx2 pathway (Wu et al., 2018). H19 is also involved in controlling MSCs’ fate, confronting nanotopographical cues of niches as the alignment of fibers *via* the BMP signaling pathway (Izadpanahi et al., 2018). During mechanical unloading, alternation of H19 may be credited to elevated DNMT1 expression. Three to four weeks of stimulated unloading resulted in an abnormally increased profile and higher nucleic-to-cytoplastic location ratio of DNMT1, causing 5 mC enrichment at CpGs in the promoter region of H19. Hypermethylation of the promoter results in lower levels of H19, followed by inhibited phosphorylation and activation of ERK and impact on TGF-β, WNT, and JAK-STAT pathways, resulting in microgravity (MG)-induced bone loss (Li et al., 2018). Another study confirmed that H19 may serve as the initiation of DOP *via* Dkk4, controlling the GSK phosphorylation ratio, which impacts the Wnt signaling pathway (Li et al., 2017). Our work, promisingly, could suggest possible network they function by and boost further studies. Besides these two well-studied lncRNAs, various creditable FR pathways were also highlighted for lncRNA identification. For instance, the RhoA/ROCK pathway is activated as a linker for the extracellular matrix to affect cellular processes, concerning the rearrangement of cytoskeleton fibers and following cell morphology changes. As an upper-stream regulator, RhoA would facilitate Smad1/5/8 phosphorylation (Ji et al., 2014) and activate YAP/TAZ (Stanley et al., 2019) while inhibiting AKT phosphorylation (Teramura et al., 2012). Significantly, YAP/TAZ stands out as the master integrator conducting the mechanic information. YAP/TAZ would shuttle from the cytoplasm to the nucleus for its binding with the transcriptional enhanced associate domain (TEAD) once sensing the signals from FA, actomyosin cytoskeleton, membrane channels, and the classic Hippo/LATS1/2 kinase cascade (Panciera et al., 2017). Furthermore, pathways such as the JUN/p38 MAPK, BMP/Smad1/5/8, TGF-β/Smad2/3, and canonical and noncanonical Wnt pathways, which are main factors involved in osteogenesis, embody the intersection with YAP/TAZ for mechanical force conduction (Azzolin et al., 2012; Gao et al., 2016). Taken together, the pathways themselves contribute to a network. Thus, we dug into these pathways to find the evidence concerning the roles of lncRNAs in Table 3. Specifically, lncRNAs like MALAT1 and NORAD, are found to have a perplexing interaction with YAP in diversified processes (Ventura, 2016; Gu L. et al., 2019; Jin et al., 2019; Tan et al., 2019; Yao et al., 2019). Besides, NORAD is highly involved with the TGF-*β*/Smad pathway direct mRNA or miRNA targeting (Kawasaki et al., 2018; Yang et al., 2019; Zhou et al., 2019). LINC00662, SNHG16, and SNHG1 are entwined with the Wnt pathway (Christensen et al., 2016; Wu W. et al., 2020; Tian et al., 2020). SNHG1, binding to EZH2, would also impact the PI3K/AKT/mTOR (Chen et al., 2020) as well as ROCK1 pathway via miR-101-3p (Deng et al., 2019). Though these models represent a close interaction of lncRNAs and key pathways in cancerous background, which is the most generalized model for lncRNA research, it could also stand as a testimony for their promising relationship in FR environments.

Clinically, essential roles of FS lncRNAs in skeletal mechanobiology as well as their therapeutic application potential could not be overlooked. Generally, MG, tensile, and compressive forces are most utilized as mechanical stimuli, mimicking varied physiological and pathological conditions. Firstly, MG implies, for the study of disuse osteoporosis (DOP), a common bone disorder when patients are in a long-term-immobilization condition like fracture, therapeutic bed rest, and space flight. H19 was found to be downregulated in the MG condition and responsible for disuse-induced bone loss (Li et al., 2017; Li et al., 2018). Its overexpression turns out to be a remedy for postmenopausal osteoporosis considering its potent effect on miR-19b-3p inhibition (Xiaoling et al., 2020). Alongside that, in a bone-related disease such as delayed fracture healing, which is characterized by the impaired ability of bone regeneration, reduced expression of lncRNA KCNQ1OT1 was suggested to be a potential biomarker while the overexpression of this lncRNA would be a possible therapeutic strategy (Gao X. et al., 2018). Novel lncRNAs such as ODSM and OGRU are also recognized as MG-sensitive ones, whose promising therapeutic roles in osteoporosis were confirmed both *in vitro* and *in vivo* (Wang M et al., 2020; Wang Y et al., 2020). While mechanical disuse would impair bone quality, muscle atrophy also represents a concern under MG circumstances. Besides, muscle–bone crosstalk against disuse condition was verified, and muscle loss would also cause bone loss (Bettis et al., 2018). Inspiringly, enforced expression of skeletal muscle-specific and MG-sensitive lncRNA, such as lncMUMA, would not only prevent muscle atrophic development but also ameliorate and even reverse the established atrophy both *in vivo* and *in vitro* (Zhang et al., 2018). Other than mechanical unloading, compressive and tensile forces contribute to bone remodeling during orthodontic tooth movement (OTM) progress, where bone resorption and formation are kept in a delicate balance. Axiomatically, minor changes induced by ncRNAs would greatly change the inclination, indicating the possibility of ncRNA modification for complication avoidance and improving efficacy of therapy. DANCR and H19 were both proved to favor osteogenesis when cells were exposed to strain force (Wu et al., 2018; Zhang X. et al., 2019). Depletion of DANCR could hinder osteoclast formation and ameliorate root resorption induced by CF both *in vitro* and *in vivo* (Zhang X. et al., 2019). Upregulated exosomal H19 of umbilical MSCs (U-MSCs), induced by an appropriate rotary cell culture condition mimicking a tensile force, exert significant remedial effects in promoting chondral regeneration and pain relieving (Yan et al., 2021). However, though adequate loading serves as a positive factor for bone homeostasis, overuse or hypertension would cause undesirable outcomes like osteoarthritis (OA). LncRNA-MSR and lncRNA-CIR, activated by excessive mechanical stress, would mediate consequent cytoskeleton disorganization and ECM degradation (Liu et al., 2016). Nonetheless, as implant and bioengineering gain popularity, the topography of the matrix and physical properties of the cell culturing environment will begin to attract researchers’ sights. MEG3 and H19 are involved in controlling MSCs’ fate in confronting nanotopographical cues of niches as a way of fiber alignment (Izadpanahi et al., 2018). Upregulated lncRNA PWRN1-209 in hBMSCs exposed to a Ti surface with microtopography would promote osteogenesis via integrin-FAK-ALP signaling (Wang M et al., 2020). Extrinsic force, like MG, CF, and strain, and intrinsic force like topography and matrix stiffness serve as essential factors for bone maintenance, and illustrating ncRNAs’ role in modulation would favor the development of tissue engineering and clinical therapy. More importantly, network analysis could greatly promote the study of skeletal diseases, considering their dynamic properties and intricate pathogenesis.

Bioinformatics has gained popularity in recent years. Though *in silico* analysis could pale in comparison with biological experiments, its strength in data processing and network analysis is impeccable. Meanwhile, growing evidences showed that predicted data of lncRNAs concerning mechanobiology could be observed in biological systems. For instance, based on the expression profile of mouse cementoblasts under static compressive force gained from sequencing, H. Liu et al. (2019) selected lncRNAs such as Prkcz2, Hklos, Trp53cor1, Gdap10, and Ak312-ps for validation, and results of RT-qPCR highly match those of sequencing data. The research group also applied compressive force to hPDLSCs (Huang et al., 2019), where RT-qPCR also validated the microarray data of FS lncRNAs such as FER1L4, HIF1A-AS2, MIAT, NEAT1, ADAMTS9-AS2, LUCAT1, MIR31HG, and DHFRP1. Moreover, upregulated FER1L4 was later proven to facilitate the compression-induced autophagy via AKT/FOXO3 as predicted (Huang et al., 2021). However, previous studies concerning lncRNAs’ role in a mechanosensitive way mainly depended on the expression profile by microarray or high-throughput sequencing (Zhang et al., 2017; Lv et al., 2018; Huang et al., 2019; Wang H et al., 2020). A limited scope and confined force type should be considered among these studies. While creating a general work of force condition to provide an overview of FR lncRNAs, we also adopted diversified strategies to guarantee and emphasize the reliability of our bioinformatic research. First, we developed a strict inclusion criteria of data and adopted prediction strategies of high stringency. The intersection of DE mRNAs under different mechanical forces contributed to the reliable identification of the FS mRNAs, and paper-based inclusion of CFS miRNAs ruled out false-positive results which usually appeared in miRNA sequencing (Iwawaki et al., 2015; Qin et al., 2019). Moreover, the predicted pairs of mRNA–miRNA were verified in all three datasets based on different computational algorithms, while lncRNA was only included when it met the standard of acquiring a highly stringent pair relationship with CFS miRNA and a degree of no fewer than three in the ceRNA network. Last, systematic analysis of lncRNAs, including the upstream factors such as TFs, the downstream pathway-mapping strategy, and the combinative analysis against FS lncRNAs, would in turn corroborate our results. Axiomatically, drawbacks exist in our work. Despite all the efforts to ensure confidence level, the flaw of bioinformatic analysis is that uncertainty persists. Besides, when our scope was expanded to all mechanical force types and included diversified bone-related cells, mechanisms specialized by cells or mechanical cues were overlooked. Certainly, though the intricate network could provide us with a blueprint, the whole network may not be activated in all mechanical situation, and factors involved under specific conditions should be further confirmed. Undeniably, concerted efforts with studies in the future would be helpful to provide further validation to our work.

Unequivocally, identification of lncRNAs needs a case-by-case basis, yet a systematic illustration is just as important, if not more so, as to provide a stepping stone to uncovering the panoramic view of this specific type of ncRNA. Though a question mark still hangs over the world of lncRNAs against mechanic cues, our work, hopefully, would produce a moment of clarity to break this impasse.

## Data Availability

The original contributions presented in the study are included in the article, further inquiries can be directed to the corresponding authors.

## Abbreviations

CTS, cyclic tensile strain; CFS miRNA, creditable force-sensitive miRNA; FS mRNA, force-sensitive mRNA; FTS mRNAs, force type sensitive mRNAs; FR lncRNAs, force-related lncRNAs; FR ceRNA, force-related ceRNA; FS lncRNA, force-sensitive lncRNA; TFBSs, transcriptional factor binding sites.
